# Evaluation of trace calls by Xpert MTB/RIF ultra for clinical management in low TB burden settings

**DOI:** 10.1371/journal.pone.0272997

**Published:** 2022-08-12

**Authors:** Alberto Amedeo, Giacomo Beci, Maddalena Giglia, Giulia Lombardi, Francesco Bisognin, Federico Chiarucci, Ilaria Corsini, Paola Dal Monte, Marina Tadolini

**Affiliations:** 1 Infectious Diseases Unit, IRCCS Azienda Ospedaliero-Universitaria di Bologna, Bologna, Italy; 2 Department of Medical and Surgical Sciences, Alma Mater Studiorum University of Bologna, Bologna, Italy; 3 Microbiology Unit, IRCCS Azienda Ospedaliero-Universitaria di Bologna, Bologna, Italy; 4 Department of Experimental, Diagnostic and Specialty Medicine–Alma Mater Studiorum University of Bologna, Bologna, Italy; 5 Department of Biomedical and Neuromotor Sciences, Section of Anatomic Pathology, Bellaria Hospital, Alma Mater Studiorum University of Bologna, Bologna, Italy; 6 Pediatric Emergency Unit, IRCCS Azienda Ospedaliero-Universitaria di Bologna, Bologna, Italy; Rutgers University, UNITED STATES

## Abstract

**Background:**

Clinical interpretation of trace results by Xpert MTB/RIF Ultra assay (Ultra) used as an initial diagnostic test for tuberculosis (TB) may be challenging. The aim of the study was to evaluate the frequency and epidemiology of trace readouts in routine clinical practice in a low TB prevalence setting and to propose guidance on how to manage patients with trace calls considering the data available (clinical, radiological, bacteriological etc.).

**Materials and methods:**

A retrospective, observational, monocentric study was conducted at IRCCS Azienda Ospedaliero-Universitaria of Bologna, Italy between November 2017—December 2020. Presumptive TB patients with at least one Ultra trace result during diagnostic workup before treatment were included in the study. Patients with ongoing anti-TB treatment at the time of the trace call result or with no clinical data available were excluded from the study.

**Results:**

Fifty-nine presumptive TB patients with Ultra trace readouts were included in the study (mean age 37.0 years, 61% males). Four patients had a history of TB in the last 2 years. Twenty-five (42.4%) of the 59 samples with trace results were respiratory material. 57/59 (96.6%) patients started anti-TB treatment soon after obtaining trace results, based on clinical, radiological or other information available, while for two patients with a recent history of TB the trace result did not lead to anti-TB treatment. Culture was positive for *M*. *tuberculosis* for 31/59 (52.5%) samples with trace calls: 13/25 (52.0%) were respiratory samples and 18/33 (54.5%) non-respiratory samples. The clinical and/or radiological findings of 47/57 (82.4%) patients given anti-TB therapy improved during treatment.

**Conclusion:**

In low TB incidence settings, Ultra trace calls in presumptive TB patients should be considered as true-positive and treatment should be started promptly, except in cases of recent history of TB, where careful evaluation of other diagnostic criteria is necessary before starting anti-TB treatment. A decisional algorithm for clinical management is proposed.

## Introduction

Tuberculosis (TB) remains a leading cause of illness and death with an estimated 9.9 million new cases and 1.5 million deaths in 2020, representing one of the major public health threats on a global level [1].

The Xpert MTB/RIF assay (Xpert, Cepheid, USA) is a cartridge-based automated molecular diagnostic test that can simultaneously identify *Mycobacterium tuberculosis* complex (MTB) and resistance/susceptibility to Rifampicin. The new version Xpert MTB/RIF Ultra (Ultra) has been developed to increase sensitivity in samples with a low mycobacterial burden [2].

In Ultra assay, diagnostic sensitivity has been improved by the addition of two multicopy genes (IS1081 and IS6110), while the single copy *rpoB* gene is used solely to determine RIF resistance. The ability of Ultra to detect *M*. *tuberculosis* DNA through IS6110 and IS1081, when there are no detectable amounts of *rpoB*, have led to the introduction of a new category of results called ‘trace’. The trace readout is characterized by the detection of a very small quantity of DNA but does not provide information about the Rifampicin resistance of the strain, as the sequence coded for by the *rpoB* gene is not detected. Therefore, additional investigations are needed to confirm or exclude resistance to Rifampicin. The other categories, defined as ‘high’, ‘medium’, ‘low’ and ‘very low’, are semi-quantitative results used by both Xpert and Ultra. They positively correlate with acid-fast bacilli detection (smear microscopy), helping to evaluate a patient’s potential to transmit TB [3, 4].

While the limit of detection has been decreased to 15.6 CFU/ml compared to 115 CFU/ml of the previous version, the new feature and trace category have reduced the test’s specificity [5–7].

It is currently unclear how the trace category should be interpreted in clinical practice. If it is considered a true-positive result and anti-TB treatment is started immediately the patient risks taking unnecessary and potentially toxic treatment. On the other hand, waiting for bacteriological confirmation from a positive Ultra on a different sample and/or MTB positive culture could mean missing, or delaying, the diagnosis of TB.

According to WHO, Ultra trace results are considered bacteriological confirmation of TB for: people living with HIV (PLHIV), children being evaluated for pulmonary TB, individuals being evaluated for extra-pulmonary TB, and adults being evaluated for pulmonary TB who are not at risk of HIV and who have not taken anti-TB treatment in the last 5 years. For HIV-negative (or not at risk of HIV) symptomatic adult patients with a recent history of treatment (i.e. completed < 5 years ago), Ultra trace results may be positive due to the presence of non-viable bacilli, rather than active TB. Therefore, clinical decisions must be based on the information available and clinical judgment. However, WHO does not recommend repeating Ultra testing in adults who have an initial Ultra trace readout to confirm the result [8].

TB prevalence strongly influences test specificity: a previous history of tuberculosis is more likely to occur in the setting of medium to high TB prevalence than in a low TB burden setting [9]. Several studies have investigated the clinical evaluation of Ultra trace results in low TB burden settings and although the introduction of this semi-quantitative category represents an important improvement to the previous Xpert version, clinical management of patients with trace calls is still challenging [3, 10–12].

The aim of the study was to evaluate the frequency and epidemiology of trace readouts in routine clinical practice in a low prevalence high-resource setting, to describe clinical management of these patients in a real-life scenario and to propose guidance on how to manage these patients considering their clinical data and other supporting evidence.

## Materials and methods

### Study design

This is a retrospective, observational, monocentric study performed at IRCCS Azienda Ospedaliero-Universitaria of Bologna, referral centre for the diagnosis and management of TB for the metropolitan area of Bologna (Emilia-Romagna Region, Northern Italy) between November 2017—December 2020.

#### Inclusion criteria

Presumptive TB; at least one Ultra trace call on biological samples during diagnostic work-up.

#### Exclusion criteria

Ongoing anti-TB treatment at the time of trace sample collection; no clinical data available.

#### Definition of presumptive TB

At least one WHO defined symptom suggestive of TB (cough, fever, night sweats, loss of body weight) and/or radiological signs suggestive of TB.

#### Paediatric population

Children under 15 years old.

The study was approved by the Ethics Committee of Area Vasta Emilia Centro (AVEC), Bologna, Italy (Protocol code n. 616/2021/Oss/AOUBo for adults and Protocol code n. 254/2018/Oss/AOUBo for children). Written informed consent was obtained from adults and from parents or guardians in case of minors, when traceable according to the Study Protocols.

### Sample processing

All specimens were stained for acid-fast microscopic examination using Ziehl-Neelsen stain, before sample decontamination. MTB isolation was performed as described in detail previously [13]. In particular, cavitary fluids and urine were centrifuged at 3500g x 15 mins at 4°C and the pellet was resuspended in 5 ml of sterile phosphate buffered saline (0.067 M, pH 6.8, PBS); biopsy and lymph-node samples were homogenized and resuspended in 5 ml of PBS. Then, samples were decontaminated with N-Acetyl-L-Cysteine-Sodium Hydroxide (NALC-NaOH) and resuspended in 2 ml PBS: 500 μl were inoculated onto 2 solid slant media (Lowenstein-Jensen; Heipha Diagnostika Biotest, Germany) and 500 μl into liquid culture (MGIT 960; Becton Dickinson, USA). Solid and liquid cultures were considered negative after 42 days of incubation without isolation of any *Mycobacteria*. An aliquot of 500 μl was tested with Ultra assay. Samples with a trace result were incubated for a further 42 days. Positive cultures were identified as MTB by Ultra or MGIT TBc Identification Test (Becton Dickinson). Time to positivity (TTP) was defined as the number of days from MGIT inoculation to the positive culture result using Epicenter software (Becton Dickinson).

Susceptibility of MTB isolates to first-line drugs (Isoniazid, Rifampicin, Ethambutol, Pyrazinamide) was tested by the gold standard automatic MGIT 960 system.

### Xpert Ultra procedure

Xpert MTB/RIF Ultra assay (Ultra, Cepheid, USA) was performed according to the manufacturer’s instructions [2]. Briefly, 500 μl of decontaminated sample were pre-treated with Sample Solution (containing NaOH and isopropanol) in a 1:3 ratio for 15 minutes at room temperature and poured into a single-use disposable cartridge of the GeneXpert module. The system automatically interpreted the fluorescent signals into the following categories: invalid (if PCR inhibitors were detected with amplification failure), negative or positive. Positive results were divided into 5 categories depending on bacterial load: the relationship between the *rpoB* Ct value and input CFU allows samples to be classified as ‘high’, ‘medium’, ‘low’ and ‘very low’ and to define strains as susceptible or resistant to Rifampicin depending on the detection of mutations in the *rpoB* gene, while the ‘trace’ category identifies the paucibacillary samples which are IS6110/IS1081 positive but *rpoB* negative, with indeterminate Rifampicin susceptibility [3–7].

### Additional TB diagnostic criteria

In addition to Ultra trace results the following TB diagnostic criteria were used, (i) clinical criteria: at least one WHO defined symptom suggestive of TB; (ii) radiological criteria: signs suggestive of TB at first level (chest X-ray, ultrasound) and/or second level imaging (high resolution computerized tomography (HRCT), CT scan w/o contrast enhancement, magnetic resonance imaging (MRI), or 18 FDG PET/CT, depending on disease localization); (iii) bacteriological tests: positive MTB culture on the same sample, positive Ultra and/or MTB culture on other samples; (iv) histopathological findings suggesting TB: necrotizing/non-necrotizing chronic granulomatous inflammation; (v) contact history or positive immunological test Interferon Gamma Release Assay (IGRA) (Quantiferon-TB Plus, Diasorin, Italy), used as supporting evidence of TB when other confirmatory elements were absent; (vi) good clinical response to anti-TB medication.

### Statistical analysis

Chi square test (Fisher’s exact test) was performed to compare the following: pulmonary and extra-pulmonary TB localization according to gender, origin, HIV status, previous TB history, culture and IGRA test. Furthermore it was also used to compare the rates of culture positivity and IGRA positivity in adults and children.

Cohen’s k statistic was used to assess agreement between histology and culture.

Student’s t-test (Mann Whitney test) was used to compare mean age and TTP between pulmonary and extra-pulmonary localization, and to compare the days before starting anti-TB treatment between adults and children.

Statistical analysis was performed using GraphPad Prism version 8.0.1 (USA). Statistical significance was set at P<0.05.

## Results

### Study population

From November 2017 to December 2020, a total of 5071 Ultra tests were carried out. The results of 80 samples (1.6%) could not be evaluated (error or invalid). Out of 4991 samples with a valid test result, 4579 (91.7%) were negative, and 412 (8.2%) were positive, with the following semi-quantitative results: 59 (14.3%) high, 79 (19.2%) medium, 133 (32.3%) low, 69 (16.7%) very low and 72 (17.5%) trace.

The 72 trace calls came from 63 patients, as 9 patients had two trace results from the same sample. 3 of these patients were excluded because clinical data were not available at the time of the study and one was excluded because the trace sample was collected during anti-TB therapy. Therefore, 59 patients were included in the analysis.

The patients’ characteristics are reported in Table 1. The mean age was 37.0 ± 17.6 years (range 1–87) and 36 patients (61.0%) were male. Nineteen patients (32.2%) came from Europe, 19 (32.2%) from Asia, 19 (32.2%) from Africa, and 2 (3.4%) from South America. Most patients (n = 56, 94.9%) were HIV-negative, 2 (3.4%) were HIV-positive and 1 (1.7%) did not perform the HIV test.

**Table 1:** Demographic and clinical characteristics of the study population.

Demographic/Clinical data	All (n = 59)	Respiratory samples (n = 25)	Non-respiratory sample (n = 34)	P-value
**Gender**				ns
Male (%)	36 (61.0)	17 (68.0)	19 (55.9)	
Female (%)	23 (39.0)	8 (32.0)	15 (44.1)	
**Mean age**				ns
years [range]	37.0 [1–87]	36.3 [1–87]	37.6 [13–73]	
**Country of origin**				ns
Native (%)	10 (16.9)	6 (24.0)	4 (11.8)	
Foreign born (%)	49 (83.1)	19 (76.0)	30 (88.2)	
**PLHIV**				
Yes (%)	2 (3.4)	1 (4.0)	1 (2.9)	ns
No (%)	56 (94.9)	23 (92.0)	33 (97.1)	
Unknown (%)	1 (1.7)	1 (4.0)	0	
**Recent TB history**				ns
Yes (%)	4 (6.8)	3 (12.0)	1 (2.9)	
No (%)	55 (93.2)	22 (88.0)	33 (97.1)	
**Culture result**				ns
Positive (%)	31 (52.5)	13 (52.0)	18 (53.0)	
Negative (%)	27 (45.8)	12 (48.0)	15 (44.1)	
Not performed (%)	1 (1.7)	0	1 (2.9)	
**IGRA test (n = 51)**		(n = 20)	(n = 31)	ns
Positive (%)	44 (86.3)	18 (90.0)	26 (83.9)	
Negative (%)	5 (9.8)	1 (5.0)	4 (12.9)	
Indeterminate (%)	2 (3.9)	1 (5.0)	1 (3.2)	

ns: not significant; PLHIV: people living with HIV; IGRA: Interferon Gamma Release Assay

The IGRA test was performed in 51 patients: 44 (82.3%) had a positive IGRA result, 5 (9.8%) negative, and 2 (3.9%) had an indeterminate result.

No significant differences were observed between pulmonary and extra-pulmonary TB localization according to gender, origin, HIV status, previous TB history, culture and IGRA results.

### Previous history of TB

Fifty-four patients (91.5%) did not report a previous history of TB, while five patients had been treated for TB in the past. One of them had been treated 9 years earlier, while the other 4 had been successfully treated in the last two years (hereafter considered as ‘recent history of TB’). The mean time since the beginning of recent treatment was 16 months (SD 3.8 months).

### Clinical presentation

All patients (n = 59) had either TB suggestive symptoms and/or radiological findings consistent with TB, meeting the definition of presumptive TB: 49 had both symptoms and radiological signs of TB, 8 had radiological signs only and 2 had TB suggestive symptoms only, with no evidence of active TB at second level chest imaging.

45/59 (76.2%) patients had first level imaging (Chest X-ray and/or ultrasound) and 55/59 (93.2%) performed second level imaging, which is standard care in our setting.

Among the patients with no recent history of TB (n = 55), 47 (85.5%) had symptoms suggestive of TB, mostly fever, productive cough and weight loss; all of them (100%) also had signs consistent with TB either at first or second level imaging. Out of these 55, 22 (40%) were investigated for presumptive pulmonary TB, and 33 for presumptive extra-pulmonary TB.

The four patients with a recent history of TB presented with TB suggestive symptoms (4/4) but only two also had imaging consistent with active TB. Three of them submitted sputum (for pulmonary TB work-up) and one underwent lymph node biopsy for extra-pulmonary TB work-up.

### Ultra trace samples

Out of the 59 samples with trace results, 25 (42.4%) were respiratory materials: 12 (48.0%) bronchoalveolar lavage (BAL), 9 (36.0%) sputum samples and 4 (16.0%) gastric aspirates. The remaining 34 (57.6%) were non-respiratory samples: 19 (55.9%) lymph node, 4 (11.8%) bone or soft tissues, 3 (8.8%) cavitary fluid, 2 (5.9%) pulmonary biopsy, 2 (5.9%) peritoneum biopsy (one peritoneum biopsy could not be cultured because it was embedded in paraffin), 2 (5.9%) urine, 1 (2.9%) salivary gland, 1 (2.9%) pleural biopsy.

All trace samples were smear negative.

### MTB culture results of Ultra trace samples

31 (53.4%) of the 58 cultured samples with trace calls were positive for MTB culture (Table 2). They comprised 13/25 (52.0%) respiratory samples and 18/33 (54.5%) non-respiratory samples (p = ns). Eleven of the 18 (61.1%) culture-positive non-respiratory samples were lymph node biopsies. In liquid culture respiratory samples had a mean time to positivity (TTP) of 19.8±5.7 days, and non-respiratory samples 19.6±8.2 days (p = ns).

**Table 2:** Culture results of Ultra trace samples by type of specimen.

Trace sample Localization	Total	Culture positive (%)
**Respiratory samples**	**25**	**13 (52.0%)**
Bronchoalveolar lavage	12	6 (50.0%)
Sputum	9	4 (44.4%)
Gastric aspirate	4	3 (75.0%)
**Non-respiratory samples**	**33**	**18 (54.5%)**
Lymph node	19	11 (57.9%)
Bone or soft tissues	4	3 (75.0%)
Cavitary fluid	3	1 (33.3%)
Pulmonary biopsy	2	1 (50%)
Peritoneum biopsy	1*	0 (0.0%)
Urine	2	1 (50.0%)
Salivary gland	1	0 (0.0%)
Pleural biopsy	1	1 (100%)
**TOTAL**	**58**	**31 (53.4%)**

* One peritoneum sample was not cultured as it was embedded in paraffin.

The majority of patients (29/31, 93.5%) had first-line pan-susceptible strains of MTB, while 2 patients had strains that were resistant to at least one first-line drug. 31/55 (56.4%) of samples from patients with no recent history of TB were culture-positive, while all samples from patients with recent anti-TB treatment were culture-negative.

### Histopathology results of Ultra trace samples

Histopathology was performed on 28 (82.3%) of the 34 extra-pulmonary samples with trace results. Histopathology suggestive of TB was found in 20 (71.4%) of these samples, while the remaining 8 had either normal findings or non-specific alterations, not pathognomonic of TB.

Seven (35%) of the 20 samples with histology suggestive of TB were culture positive, as were 7/8 (87.5%) of samples with negative histology. Therefore, agreement between histology and culture results was very low (8/28 = 28.6%, k<0.01). One sample with positive histology could not be cultured because it was embedded in paraffin.

### IGRA test

Almost all patients 51/59 (86.4%) had the immunological test IGRA(Quantiferon-TB Plus). Forty-four (86.3%) were positive, 5 (9.8%) were negative and 2 (3.9%) had an indeterminate result due to low Mitogen value.

### Decision to treat

The diagnostic criteria used to support or rule out TB diagnosis in culture negative and culture positive patients are summarized in Table 3 and supplementary material S1 Table, respectively.

**Table 3:** Characteristics of patients with Xpert Ultra trace call results and culture negative samples.

Patient ID	Sex	Age	Country of origin	Sample type	Recent TB history	Suggestive symptoms	Suggestive imaging	Compatible histopathology	Other samples with Xpert and/or culture positive	IGRA	Decision to treat	Clinical (C)/Radiological (R) outcome at the end of treatment
**Respiratory samples**												
5	F	33.1	Romania	Sputum	Yes	Yes	Yes	Not done	Yes	Not done	Yes	C+R improvement
7	M	24.2	Ivory Coast	Sputum	No	No	Yes	Not done	Yes	Pos	Yes	C+R improvement
18	M	51.8	Pakistan	Sputum	Yes	Yes	No	Not done	No	Not done	No	N/A
24	M	45.2	Ukraine	Sputum	No	Yes	Yes	Not done	Yes	Pos	Yes	R improvement
30	M	78.4	Italy	Sputum	Yes	Yes	No	Not done	No	Ind	No	N/A
8	F	30.5	Morocco	BAL	No	Yes	Yes	Not done	No	Pos	Yes	C+R improvement
16	M	71.7	Italy	BAL	No	No	Yes	Not done	No	Pos	Yes	C+R improvement
21	M	27.8	Morocco	BAL	No	No	Yes	Not done	No	Pos	Yes	C+R improvement
50	F	87.4	Italy	BAL	No	Yes	Yes	Not done	No	Pos	Yes	C+R improvement
52	M	33.2	Iran	BAL	No	Yes	Yes	Not done	No	Pos	Yes	C+R improvement
58	M	25.2	Guinea	BAL	No	Yes	Yes	Not done	Yes	Pos	Yes	R improvement
6	M	1.2	Kosovo	Gastric aspirate	No	No	Yes	Not done	Yes	Pos	Yes	C+R improvement
**Non-respiratory samples**												
9	F	39.1	India	Lymph node	No	Yes	Yes	Yes	No	Pos	Yes	C+R improvement
32	F	41.0	Pakistan	Lymph node	Yes	Yes	Yes	Yes	No	Pos	Yes	C+R improvement
37	F	68.9	Romania	Lymph node	No	Yes	Yes	Yes	Yes	Pos	Yes	C+R improvement
38	M	58.0	Pakistan	Lymph node	No	Yes	Yes	Yes	No	Neg	Yes	C+R improvement
47	F	60.3	Bangladesh	Lymph node	No	Yes	Yes	No	No	Pos	Yes	Transfer out
54	M	49.9	China	Lymph node	No	Yes	Yes	Not done	No	Pos	Yes	Transfer out
56	M	51.0	Italy	Lymph node	No	Yes	Yes	Yes	Yes	Pos	Yes	C+R improvement
59	F	40.1	Tanzania	Lymph node	No	Yes	Yes	Yes	Yes	Not done	Yes	C+R improvement
13	F	27.6	Peru	Peritoneal fluid	No	Yes	Yes	Yes	No	Pos	Yes	C improvement
43	M	21.3	Guinea	Pleural fluid	No	Yes	Yes	Yes	No	Pos	Yes	C+R improvement
26	M	42.6	Morocco	Salivary gland	No	Yes	Yes	Yes	No	Pos	Yes	C+R improvement
14	M	38.5	Peru	Peritoneum biopsy	No	Yes	Yes	Yes	No	Pos	Yes	C+R improvement
33	F	28.7	Morocco	Peritoneum biopsy*	No	Yes	Yes	Yes	No	Pos	Yes	C+R improvement
17	F	43.3	Morocco	Bone biopsy	No	Yes	Yes	Yes	No	Pos	Yes	C improvement
53	F	39.4	Italy	Pulmonary biopsy	No	Yes	Yes	Yes	Yes	Not done	Yes	C+R improvement
40	M	73.4	Italy	Urine	No	Yes	Yes	Not done	No	Neg	Yes	C+R improvement

N/A: Not applicable (patient not started on treatment);

* Embedded in paraffin; Ind: indeterminate result

Fifty-seven (96.6%) patients started anti-TB treatment, based on clinical and/or radiological findings and an Ultra trace call.

Specifically, all patients with no previous history of TB (n = 54), and three patients with a history of TB (2 with recent history of TB and one with past history of TB) were started on anti-TB treatment immediately after the trace result (4.0±4.7 days after the Ultra result). Although drug susceptibility test results and Rif-resistance patterns were not available at the time of treatment initiation, all patients were started on first-line treatment, in view of the low risk of MDR-TB in our setting. Two patients with a recent history of TB were not restarted on treatment due to insufficient evidence of TB relapse: both presented with symptoms but chest HRCT findings were not suggestive of active TB (showing only TB sequelae). In their cases the trace result was therefore interpreted as a residual finding of the previous episode (false-positive). Culture was negative for all of their sputum samples.

### Response to treatment

All 57 subjects started on treatment have a treatment outcome available: 47 (82.4%) achieved treatment success with clinical and radiological improvement, 7 (12.3%) were lost to follow-up and 3 (5.3%) were transferred out.

### Paediatric population with Ultra trace results

Seven (11.9%) of the 59 patients included in the study were paediatric patients (mean age 9.7± 5.8 years, 5/7 were males), all of them belonged to families from middle/high TB burden countries. Five children had symptoms suggestive of TB and 7/7 had radiological findings consistent with TB. In four cases the trace result was obtained on gastric aspirate, in two cases from lymph node needle biopsy and one from bone biopsy.

MTB grew in culture of 6/7 (85.7%) samples, with a higher culture positivity than in adults (25/51, 49%). However this difference was not statistically significant, probably due to the limited pediatric population size.

IGRA test was performed in all children during diagnostic workup: 6/7 (85.7%) had a positive result, and one converted from negative (even if close to the cut-off, TB1 0.32 and TB2 0.29 IFN-γ IU/ml) to positive (TB1 3.44 and TB2 2.91 IFN-γ IU/ml) after 2 weeks; IGRA positivity in children was comparable to that in adults (38/44, 86.4%).

All children were started on anti-TB treatment 1±1.1 days after trace results, compared to 4.4±4.9 days in adults (p = 0.012). All children showed clinical and/or radiological improvement after anti-TB treatment.

## Discussion

Recent studies on Ultra assay in low prevalence TB settings have essentially compared the sensitivity and specificity with the previous version, Xpert [3, 10, 11]. All Authors report greater sensitivity with Ultra than Xpert, particularly in paucibacillary infections such as pulmonary smear-negative and extra-pulmonary TB. In particular, we reported elsewhere an overall improvement of 45.6% in MTB detection compared to the previous Xpert assay on Xpert negative/culture positive samples [10]. In Opota and colleagues’ study, trace readouts were only found in patients with active TB, with the limitation of small sample size [3]. In Piersimoni’s study 14/169 (8.2%) positive Ultra samples were trace calls. 4 (28.6%) of these were considered false-positive results, supported by negative culture and negative Ultra results on repeated samples [11]. In a recent multicentre evaluation study performed in Italy, 317 trace results from both respiratory and non-respiratory samples were analysed. 239 (75.4%) were considered true-positives based on both microbiological and clinical data, 25 (7.9%) had a previous history of TB and 53 (16.7%) were considered false-positives, as a diagnosis of TB was excluded or considered improbable. The proportion of true-positive results is clearly higher than the false-positives [12]. However, the clinical interpretation of trace calls remains largely unclear. In active case finding scenarios, such as recent TB prevalence surveys using Ultra, trace calls were considered negative [14].

Our study comprised 59 patients with presumptive TB who had at least one trace readout during diagnostic workup. Culture results were obviously still not available when the trace results were recorded, so other criteria had to be used to interpret the trace calls, and to decide whether to start treatment or not.

As recommended by WHO, when the Ultra test result is trace, many other factors must be taken into consideration, such as the patient’s age, HIV-infection status, history of anti-TB treatment and sample localization [8].

Our low TB incidence setting is characterized by a small number of previously treated patients. In our study the majority of patients with presumptive TB and trace calls were new cases (55/59, 93.2% had no recent history of TB) making interpretation of the trace calls somewhat easier for clinicians. In addition, all individuals with no recent history of TB, including two PLHIV, had first or second level imaging suggestive of TB. This was considered sufficient to start treatment immediately after the Ultra result, with a mean interval of 4.0 days between the trace result and treatment initiation.

In 31/55 (56.4%) of new cases, a MTB positive culture on the same sample later confirmed the diagnosis of TB. In our experience, culture positivity in trace samples (31/58, 53.4% overall) appears higher than in other studies with similar population sizes [6, 15, 16]. The possible explanations include: high quality samples (33 extra-pulmonary and 12 bronchoalveolar lavage specimens), cold-chain maintenance and rapid sample processing (within 24 hours), solid (2 tubes) and liquid culture (1 tube) performed in parallel for each sample, prolonged incubation time in cases of Ultra trace calls (up to 84 days), dedicated and well-trained laboratory staff. In particular, solid culture permitted the identification of 2 positive cultures (negative in MGIT) and prolonged MGIT incubation detected 1 culture-positive sample (growth after 47 days). Of note, culture positivity was similar for respiratory and non-respiratory samples (13/25, 52.0% in respiratory samples vs. 18/33, 54.5% in non–respiratory samples; p = ns), as previously described for Xpert MTB/RIF [13]. In culture-confirmed cases the decision to start treatment based on trace calls gained a mean of 19 days compared to culture result availability. Drug susceptibility tests on culture isolates excluded MDR-TB cases, and confirmed the appropriateness of the first-line treatment prescribed.

In 24 patients with non-positive culture (23 culture negative; 1 culture not done), diagnosis of TB was supported by histopathology (n = 12), clinical/radiological improvement under anti-TB treatment (n = 20) and culture and/or Ultra positive results from a different sample (n = 9).

Interpretation of trace calls in previously treated patients is more challenging, especially if the last episode of TB was recent (<2–5 years). In our study the decision to re-treat the only patient with a past history of anti-TB treatment (9 years earlier) was based on clinical/radiological findings and the trace result, as detection of the MTB genome is unlikely after such a long period of time. Culture positivity subsequently confirmed the presence of active TB disease. In contrast, only two of the four patients with a recent history of successful anti-TB treatment, were restarted on treatment, based on clinical/radiological findings in addition to the trace result, and both of them responded well to treatment. The trace calls of the remaining two were considered false-positives related to the previous episodes, as there were not enough elements to support a diagnosis of active disease (radiology not suggestive of active TB). The two patients who were not treated based on trace calls were monitored for 6 and 12 months; they did not show any progression to active TB over time.

When deciding whether to treat individuals with presumptive TB based on trace readouts (in the absence of culture results), there are other elements that should be taken into consideration to corroborate the diagnosis of active TB: clinical presentation, detailed imaging (i.e. CT scan, MRI, PET or others) in addition to radiography or ultrasound, and histopathology findings (especially for extra-pulmonary TB localizations). As far as previous treatment history is concerned, not only the timing of treatment, but also treatment outcome is crucial for deciding how to manage the patient.

**Fig 1:**
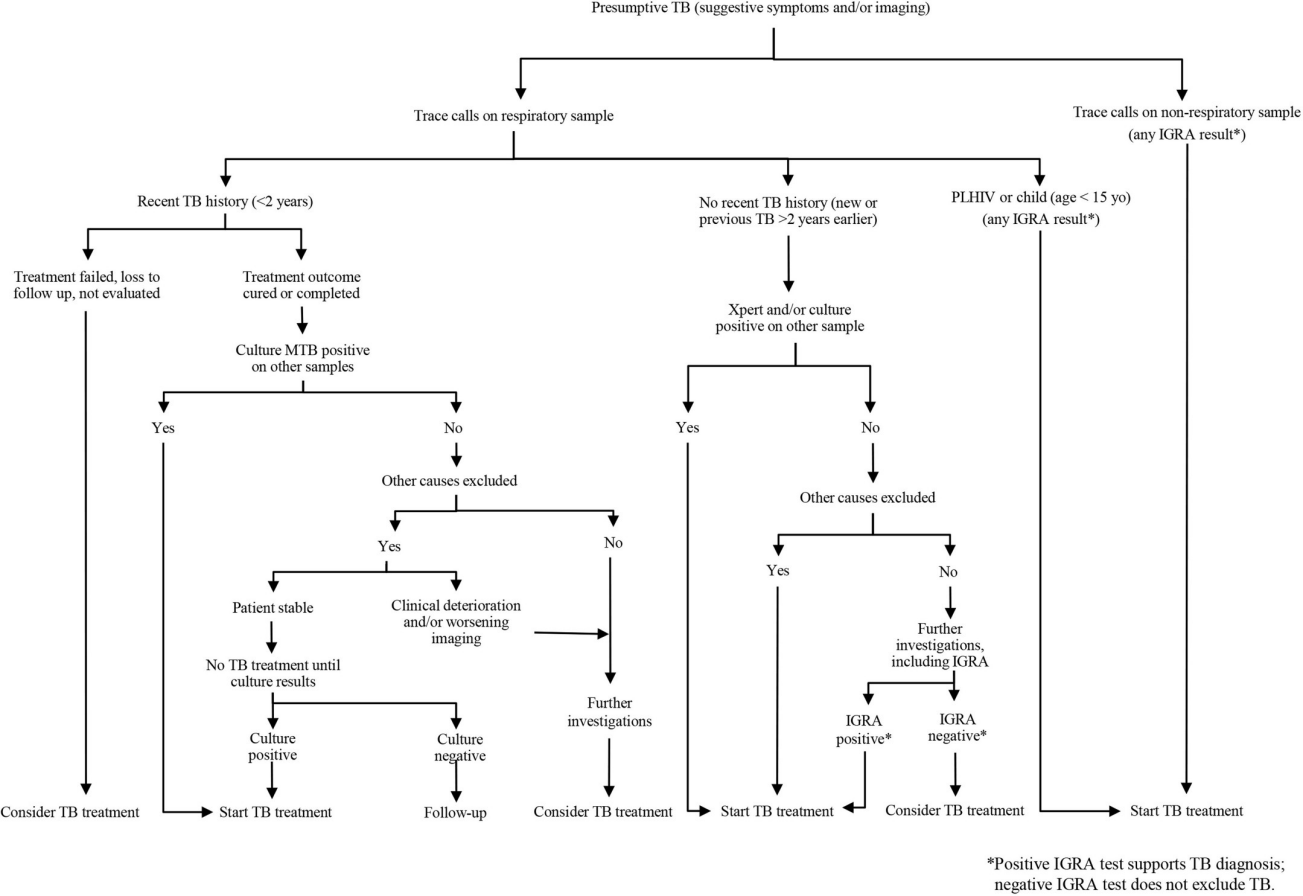
Proposed diagnostic algorithm for the management of presumptive TB patients with Ultra trace results.

Based on our experience, we propose using the following diagnostic algorithm (summarized in Fig 1). In patients with presumptive TB and a trace call on biological samples, diagnosis of TB can be formulated (and anti-TB treatment started) in the following scenarios:

- Trace result on non-respiratory sample (extra-pulmonary disease localization);
- Paediatric population;
- PLHIV;
- Positive Ultra on a different sample in patients with no recent history of TB;
- Suggestive histopathology and exclusion of other causes;
- MTB positive culture (on the same sample with trace call or a different sample), regardless of TB treatment history;
- Clinical deterioration and/or radiological progression in patients with recent successful treatment history and no other elements available (and other causes excluded).

Positive IGRA cannot be considered confirmatory evidence of TB, but in the paediatric population and when other criteria are lacking, a positive IGRA test can support the diagnosis of TB in low incidence settings. In particular, a positive IGRA test in children with symptoms and/or signs of TB is highly suggestive, considering that TB infection in childhood in low burden settings is unlikely. Last but not least, a favourable clinical and/or radiological response to anti-TB treatment corroborates the diagnosis of TB at a later stage (ex-juvantibus diagnosis).

In cases of recent anti-TB treatment with negative outcome (treatment failure, lost to follow-up, not evaluated), re-starting anti-TB treatment based on trace calls can be considered. However, in these cases, further drug susceptibility tests would be helpful.

Our study has some limitations; the retrospective nature, the limited size of the cohort, and the small number of patients with HIV infection (2/59) or a recent history of anti-TB treatment (only 4/59). Therefore, it is not possible to draw conclusions about the significance of trace calls in previously-treated TB patients. However, as already mentioned elsewhere [17], trace calls in patients with a recent history of anti-TB treatment need careful evaluation; new anti-TB treatment should be considered only if there are other elements suggestive of active TB (clinical worsening, active TB suggestive imaging, positive MTB culture).

In conclusion, based on our experience, trace calls in presumptive TB patients with no recent history of TB in a low incidence setting should be considered true-positives and should lead to treatment initiation. In the absence of a Rif-susceptibility pattern, selection of the treatment regimen should be based on epidemiological and individual MDR-TB risk factors. On the contrary, the interpretation of trace calls in recently treated patients requires careful evaluation of other diagnostic criteria before starting anti-TB treatment. A larger multi-centre study could help to confirm our results.

## Supporting information

S1 Table Characteristics of patients with Ultra trace and positive culture by sample type. (DOC)
