# Supplementary material for: Evaluation of trace calls by Xpert MTB/RIF ultra for clinical management in low TB burden settings
Source: PLoS One. 2022 Aug 12;17(8):e0272997. doi: 10.1371/journal.pone.0272997 (PMC9374243; doi:10.1371/journal.pone.0272997)
Supplement: S1 Table — (DOC) [file pone.0272997.s001.doc]

| **Patient**  **ID** | **Sex** | **Age** | **Country of origin** | | | **Sample type** | | | **Recent TB history** | | | **Suggestive symptoms** | | | **Suggestive**  **imaging** | | | **Compatible histopathology** | | | **Other samples with Xpert and/or culture positive** | | | **IGRA** | | **Decision**  **to treat** | **Clinical (C)/**  **Radiological (R) outcome at the end of treatment** | | |
| --- | --- | --- | --- | --- | --- | --- | --- | --- | --- | --- | --- | --- | --- | --- | --- | --- | --- | --- | --- | --- | --- | --- | --- | --- | --- | --- | --- | --- | --- |
| **Respiratory samples** | | | | |  | | |  | | |  | |  | | | |  | |  | | |  | |  | | |  | |  |
| 3 | F | 21.3 | Nigeria | | | Sputum | | | No | | | Yes | | | Yes | | | Not done | | | No | | | Not done | | Yes | lost to follow-up | | |
| 22 | F | 44.8 | Romania | | | Sputum | | | No | | | Yes | | | Yes | | | Not done | | | Yes | | | Pos | | Yes | C improvement | | |
| 42 | M | 40.2 | Romania | | | Sputum | | | No | | | Yes | | | Yes | | | Not done | | | No | | | Not done | | Yes | lost to follow-up | | |
| 46 | M | 25.9 | Bangladesh | | | Sputum | | | No | | | Yes | | | Yes | | | Not done | | | No | | | Pos | | Yes | lost to follow-up | | |
| 2 | F | 33.3 | Moldova | | | BAL | | | No | | | No | | | Yes | | | Not done | | | No | | | Not done | | Yes | transfer out | | |
| 4 | M | 27.9 | Pakistan | | | BAL | | | No | | | No | | | Yes | | | Not done | | | No | | | Pos | | Yes | C+R improvement | | |
| 34 | M | 44.0 | Pakistan | | | BAL | | | No | | | Yes | | | Yes | | | Not done | | | No | | | Pos | | Yes | C+R improvement | | |
| 48 | F | 55.2 | Italy | | | BAL | | | No | | | Yes | | | Yes | | | Not done | | | No | | | Pos | | Yes | C+R improvement | | |
| 51 | F | 37.6 | Pakistan | | | BAL | | | No | | | Yes | | | Yes | | | Not done | | | Yes | | | Pos | | Yes | C+R improvement | | |
| 57 | M | 51.3 | Italy | | | BAL | | | No | | | Yes | | | Yes | | | Not done | | | No | | | Pos | | Yes | C+R improvement | | |
| 31 | M | 14.2 | Pakistan | | | Gastric aspirate | | | No | | | No | | | Yes | | | Not done | | | Yes | | | Pos | | Yes | C+R improvement | | |
| 35 | M | 2.9 | Germany | | | Gastric aspirate | | | No | | | Yes | | | Yes | | | Not done | | | No | | | Pos | | Yes | lost to follow-up | | |
| 44 | M | 10.5 | Italy | | | Gastric aspirate | | | No | | | Yes | | | Yes | | | Not done | | | No | | | Neg | | Yes | C+R improvement | | |
| **Non-respiratory samples** | | | |  | | |  | | |  | | | |  | |  | | | |  | | |  | |  | | |  | |
| 1 | F | 30.0 | Pakistan | | | Lymph node | | | No | | | Yes | | | Yes | | | Not done | | | Yes | | | Pos | | Yes | C+R improvement | | |
| 11 | F | 14.8 | Eritrea | | | Lymph node | | | No | | | Yes | | | Yes | | | No | | | Yes | | | Pos | | Yes | C+R improvement | | |
| 12 | F | 34.1 | Morocco | | | Lymph node | | | No | | | Yes | | | Yes | | | No | | | No | | | Not done | | Yes | C improvement | | |
| 15 | M | 37.8 | Pakistan | | | Lymph node | | | No | | | Yes | | | Yes | | | Yes | | | Yes | | | Pos | | Yes | lost to follow-up | | |
| 20 | M | 20.8 | Senegal | | | Lymph node | | | No | | | No | | | Yes | | | No | | | No | | | Pos | | Yes | lost to follow-up | | |
| 25 | M | 39.4 | Congo | | | Lymph node | | | No | | | Yes | | | Yes | | | Yes | | | No | | | Pos | | Yes | C+R improvement | | |
| 27 | F | 14.8 | Italy | | | Lymph node | | | No | | | Yes | | | Yes | | | No | | | Yes | | | Pos | | Yes | lost to follow-up | | |
| 28 | F | 45.6 | Pakistan | | | Lymph node | | | No | | | Yes | | | Yes | | | No | | | No | | | Pos | | Yes | C+R improvement | | |
| 29 | M | 40.3 | Pakistan | | | Lymph node | | | No | | | Yes | | | Yes | | | Yes | | | No | | | Pos | | Yes | C improvement | | |
| 41 | F | 26.4 | Capo Verde | | | Lymph node | | | No | | | Yes | | | Yes | | | No | | | No | | | Pos | | Yes | C+R improvement | | |
| 45 | M | 24.3 | Senegal | | | Lymph node | | | No | | | Yes | | | Yes | | | Yes | | | No | | | Neg | | Yes | C+R improvement | | |
| 19 | M | 40.0 | Pakistan | | | Bone biopsy | | | No | | | Yes | | | Yes | | | Yes | | | Yes | | | Neg | | Yes | C+R improvement | | |
| 23 | M | 13.6 | Nigeria | | | Bone biopsy | | | No | | | Yes | | | Yes | | | Not done | | | Yes | | | Pos | | Yes | C+R improvement | | |
| 49 | M | 28.4 | Somalia | | | Bone biopsy | | | No | | | Yes | | | Yes | | | Not done | | | Yes | | | Pos | | Yes | C+R improvement | | |
| 10 | M | 50.5 | Philippines | | | Pulmonary biopsy | | | No | | | Yes | | | Yes | | | No | | | Yes | | | Pos | | Yes | C+R improvement | | |
| 36 | M | 28.4 | Sri Lanka | | | Pleural biopsy | | | No | | | Yes | | | Yes | | | Yes | | | Yes | | | Pos | | Yes | C+R improvement | | |
| 55 | M | 49.5 | Romania | | | Pleural fluid | | | No | | | Yes | | | Yes | | | Not done | | | Yes | | | Ind | | Yes | C+R improvement | | |
| 39 | M | 23.6 | Sierra Leone | | | Urine | | | No | | | Yes | | | Yes | | | Yes | | | Yes | | | Pos | | Yes | C+R improvement | | |

**Table S1 Characteristics of patients with Ultra trace and positive culture by sample type.**
